# Towards an End-to-End Framework of CCTV-Based Urban Traffic Volume Detection and Prediction

**DOI:** 10.3390/s21020629

**Published:** 2021-01-18

**Authors:** Maria V. Peppa, Tom Komar, Wen Xiao, Phil James, Craig Robson, Jin Xing, Stuart Barr

**Affiliations:** 1 School of Engineering, Newcastle University, Newcastle upon Tyne NE1 7RU, UK; maria-valasia.peppa@ncl.ac.uk (M.V.P.); craig.robson1@ncl.ac.uk (C.R.); jin.xing@ncl.ac.uk (J.X.); 2 Urban Observatory, Newcastle University, Newcastle upon Tyne NE1 7RU, UK; tom.komar@ncl.ac.uk (T.K.); philip.james@ncl.ac.uk (P.J.); 3 Australian Urban Research Infrastructure Network (AURIN), The University of Melbourne, Corner Swanston and Elgin Street, Perth, VIC 3010, Australia; stuart.barr@unimelb.edu.au

**Keywords:** traffic prediction, deep learning, intelligent transportation systems, IoT, geospatial data

## Abstract

Near real-time urban traffic analysis and prediction are paramount for effective intelligent transport systems. Whilst there is a plethora of research on advanced approaches to study traffic recently, only one-third of them has focused on urban arterials. A ready-to-use framework to support decision making in local traffic bureaus using largely available IoT sensors, especially CCTV, is yet to be developed. This study presents an end-to-end urban traffic volume detection and prediction framework using CCTV image series. The framework incorporates a novel Faster R-CNN to generate vehicle counts and quantify traffic conditions. Then it investigates the performance of a statistical-based model (SARIMAX), a machine learning (random forest; RF) and a deep learning (LSTM) model to predict traffic volume 30 min in the future. Tests at six locations with varying traffic conditions under different lengths of past time series are used to train the prediction models. RF and LSTM provided the most accurate predictions, with RF being faster than LSTM. The developed framework has been successfully applied to fill data gaps under adverse weather conditions when data are missing. It can be potentially implemented in near real time at any CCTV location and integrated into an online visualization platform.

## 1. Introduction

Near-future predictions of traffic conditions across an arterial road network have been a fundamental part of intelligent transport system (ITS) technology for a few decades now. Analyzing and predicting traffic conditions in real time can effectively support urban road traffic management, resulting in reduced road disruptions and delays, providing congestion warnings as well as allocating resources for a safe and sustainable urban infrastructure. Nowadays, ITSs operate in conjunction with the Internet of Things (IoT) [1] and big data analytics [2] for effective urban traffic management, indicating the importance of two main aspects: (a) The traffic flow or volume analysis and prediction approaches applied; and (b) the traffic sensor infrastructure installed and used. Parallel to those two aspects, computational capacity has to be considered when integrating models and sensors for real-time applications and automated IoT sensing systems.

Traffic flow analysis and prediction have been a major area of interest within the field of ITSs since the late 1970s [3]. Typically, the number of vehicles constitutes one of the main parameters to analyze urban traffic behavior, also indicative of traffic volume that is used hereafter. Additional parameters include type, height and other characteristics of a vehicle. Many approaches have been developed to extract traffic volume and other parameters from various sensors. The faster region based convolutional neural network (Faster R-CNN; [4]) has been a well-established deep learning approach used for vehicle detection and classification from images of camera sensors set up at road intersections or from aerial platforms. The study in [5] investigated the Faster R-CNN performance in vehicle detection by optimizing parameters for model fine-tuning. To run their experiments they used the KITTI open-source benchmark image datasets, developed by the Karlsruhe Institute of Technology and Toyota Technological Institute (KITTI; [6]). Another benchmark image dataset is Common Objects in Context (COCO; [7]) introduced by Microsoft. Both datasets contain images from the natural and built environment from various regions, used to train deep learning models. As a result, open to the public pre-trained frozen graph inferences have been established (e.g., in [8]). These pre-trained models have supported research on vehicle count generation and vehicle classification. However, to accurately quantify the traffic conditions across a local arterial network, datasets obtained from the network’s infrastructure are essential.

Regarding traffic prediction approaches, neural network-based methods have been the most popular ones according to the review in [9]. For instance, the study in [10] has developed a long short-term memory (LSTM) model, which is a type of advanced recurrent neural network (RNN), for predicting vehicle speeds on expressways using data from roadside loop detectors. More recently, the authors in [11] have combined multiple LSTM models with k-nearest neighbor (KNN), a traditional machine learning (ML) approach, to predict traffic using data from nearby loop detectors with high spatiotemporal correlations. Additionally, the authors of [12] developed a stacked auto-encoder model that includes multiple layers of contemporary neural networks to predict traffic on freeways. In all these studies, the authors have reported that their advanced proposed architectures outperform other simple approaches with an approximate average accuracy improvement ranging from 3% to 20%.

It is notable that the aforementioned studies [10,11,12] demonstrate exceptional examples of innovative model development. However, it is not always guaranteed that advanced deep learning models can successfully be applied to any type of traffic data. According to [13], simple architectures can sometimes work more efficiently than complex advanced methods. The latter usually demand a series of “trial and error” tests for tuning parameterization, increasing their life cycle cost [13]. The choice of prediction model is strongly dependent on the type of prediction problem and the characteristics of the traffic data used as input [13]. Nevertheless, compared to traditional ML methods, deep learning models are not easily interpretable [14], hence, expert knowledge is often required [15].

In terms of conventional traffic sensor infrastructure, numerous studies have extensively used in-ground or roadside inductive loop detectors for traffic prediction [2,12,16,17]. Other popular traffic sensors include the global navigation satellite systems (GNSSs) embedded in smartphones [18] or those installed in taxis [19]. However, the aforementioned sensor infrastructure requires a particular installation and can be relatively costly for traffic management bureaus when installed at multiple locations across an entire city or in hundreds of taxis. An alternative low-cost [2] and widely available sensor infrastructure can be closed-circuit television (CCTV) systems which have been primarily employed for traffic surveillance, vehicle detection (e.g., automatic number plate recognition (ANPR) systems; [20]) and tracking [21] as well as event recognition applications [22,23,24], but not explicitly used for traffic prediction. Compared to studies using loop detectors and GNSS sensors, relatively little research on prediction has been conducted with CCTV datasets in recent years (e.g., in [25]). In addition to this, more published research has been conducted on highways or freeways whilst urban traffic prediction is yet to be investigated fully, as only one-third of published work is focused on urban arterials, as recently reported in [9]. To facilitate such research in urban environments, CCTV datasets have recently become freely available from many local authorities in the UK through initiatives such as the Urban Observatory (UO) project in the North East of England, hosted by Newcastle University [26].

With the emergence of deep learning technology, a considerable literature has grown up primarily around the development of novel individual architectures. On the one hand, this, together with the freely available benchmark datasets (e.g., COCO) and raw sensor observations (e.g., CCTV image series), has led to the growth of numerous open-source libraries consisting of state-of-the-art object detection and time series prediction approaches (e.g., in [8,27]). On the other hand, as also discussed in a very recent study in [28], to apply deep learning approaches for real-time predictions requires high computational capacity to train and update prediction models when new real-time traffic information is retrieved. Whilst there is a plethora of advanced approaches, a seamless practical workflow for both traffic flow detection and prediction with minimal computational cost is yet to be further developed. Moreover, combined traffic detection and prediction from raw IoT sensing data would significantly benefit traffic monitoring and management, especially when used on integrated platforms where raw data, detections and predictions can be explored and visualized.

To that end, the presented research aims to develop an end-to-end automated CCTV-based traffic volume analysis and prediction framework that is computationally fast and effective to be potentially used for near real-time applications. The main motivation of the research is to take advantage of commonly available raw IoT CCTV imagery alongside advanced algorithms within an integrated pipeline (hence the term “end-to-end”) to provide a twofold outcome: (a) Quantification of urban traffic and (b) estimation of future traffic conditions. This framework is intended to support the decision-making process in a local traffic bureau for proactive actions under disruptive circumstances. Specifically, the framework incorporates state-of-the-art CNNs for generating vehicle counts as identified in CCTV image series, quantifying the arterial traffic volume conditions of the North East region, UK. It then utilizes free and open-source libraries for three models (i.e., one statistical-based model, one machine learning model and one deep learning model) to predict traffic volume at multiple locations across the North East. Tests assess the three prediction models at six locations with different lengths of historical vehicle counts and by incorporating calendar attributes as well as spatio-temporal information from other nearby CCTV cameras. Additionally, a use case of the framework is demonstrated for a six-day period to fill gaps when data are missing from the CCTV image series. The possibility of framework integration with an online demonstrator is also explored.

The main contributions of the study are as follows:

- To demonstrate the use of raw CCTV images for traffic prediction in complex urban areas within a full end-to-end framework;
- To provide constantly updated traffic volume (i.e., vehicle counts) as an open-source dataset to the general public, traffic managers and the research community;
- To develop an efficient traffic detection and prediction framework with the potential for near real-time implementation, such as integrating into a live online platform.

The remainder of the paper is organized as follows: In Section 2, related work is described. In Section 3, the methodology is presented, including the data used, the developed framework and the experiments conducted. Section 4 demonstrates the results of traffic prediction per experiment. In Section 5, a discussion of the results is presented with the future directions of the developed framework and Section 6 concludes the main findings of the work.

## 2. Related Work

### 2.1. CNNs for Vehicle Detection

Since early 1990s, CNNs have been applied in various studies, such as the recognition of handwritten numbers [29], but they have explicitly facilitated research in image processing and object recognition [14]. Deep CNNs consist of multiple neurons with learnable filters, which are activated after processing a raw input image using various convolutional operations (e.g., gradients, blobs, edge filters etc.). A structured network of neurons can localize subtle characteristics of features in a raw image using a combination of learnable variables (e.g., weights and biases) [14]. Nowadays, an advanced CNN architecture, Faster R-CNN, has been widely implemented in vehicle detection from images [5]. R-CNN segments each convolutional layer using a sliding window into proposed regions, which contain the predicted location of multiple anchor boxes of different sizes and scales per object. A global optimization refines the predicted box location with the aid of regression algorithms.

Due to the design of region proposals, Faster R-CNN outperforms in object detection compared to other CNN models, as reported in [5]. The study in [30] assessed the performance of Faster R-CNN against the single shot multibox detector (SSD) MobileNet; an alternative deep CNN specifically designed for mobile applications [31]. They found that a pre-trained Faster R-CNN ResNet model fine-tuned with 700 CCTV images could identify more small vehicles in the image background, providing better precision and fewer false negatives than the fine-tuned SSD model. To address the issue of varying vehicle scales in a natural scene, the study in [32] optimized the default anchor box size and changed the default combination of convolutional layers in the Faster R-CNN architecture using KITTI image datasets. That way, they further improved its performance in detecting small vehicles by circa 7%.

### 2.2. Traffic Prediction Approaches

Over the years, extensive reviews have categorized the numerous traffic prediction approaches based on different factors, such as the input data, the application, the methodology, etc. According to the review in [33], traffic prediction approaches can be generally categorized as naïve, parametric and nonparametric. An example of a naïve approach, applied in [34,35], included the calculation of the historical average (HA) to predict future traffic over a specified time interval. The authors in [35] also applied a naïve approach solely based on the traffic values of the previous day to compute the traffic for the following day in the same time interval. Even though naïve methods are computationally easy to implement, they rely on the past traffic behavior without additional information and therefore are limited in predicting abnormal situations [33,34].

Based on previous reviews [3,33], parametric approaches mostly include analytical and traffic simulation-based methods. In such methods, traffic flow or volume is predicted via mathematical equations derived from traffic theory [10,33]. As mentioned in [10] and [33], these methods are still widely employed as they use physical assumptions to aid in interpreting transportation functionality. However, they are often weak in modeling real-time traffic systems, especially when such systems comprise big data [10]. Other parametric approaches also include statistical-based methods, with the seasonal autoregressive integrated moving average (ARIMA; [36]) model being one of the most widely established strategies for traffic prediction [35,37,38,39]. A series of ARIMA variants, such as seasonal ARIMA (SARIMA; [39]), ARIMA with exogenous features (ARIMAX; [40]) and others, were gradually developed over the years. In theory, an ARIMA model, as a linear statistical approach, considers that the properties of a traffic time series are constant and any variations around its mean typically follow a consistent pattern [10]. As ARIMA models often mishandle the stochastic and nonlinear nature of traffic behavior, it has been superseded through the emergence of nonparametric approaches. However, ARIMA variants are still used in traffic prediction as benchmarks for comparative purposes [3] due to their ease of implementation.

Typical nonparametric approaches primarily include: Kalman filtering [41]; support vector regression (SVR) [25,42]; random forest (RF) regression [43]; and traditional artificial neural networks (ANNs) [44]. Among those, the review here is focused on RF regression. RF is a supervised ML algorithm based on decision tree models where data are sub-sampled, trained and predicted multiple times under individual well-structured trees [43,45]. A particular averaging method is applied to assess the variance and accuracy per prediction and a mean prediction is finally voted on while the algorithm converges [43]. As reported in [46], RF can handle overfitting with sufficient speed when large data series are used, compared to other similar regression tree algorithms. A more recent study in [47] reported that RF achieved the lowest average errors compared to other regression models for predicting travel times across an urban road network. An empirical analysis in [48] acknowledged the limitation that fine-tuning is a time-consuming procedure. In addition, a previous study in [49] showed that RF requires little time for tuning, favorable for real-time applications.

Enhanced nonparametric approaches primarily include recently developed deep learning RNNs. LSTM is one of the most widely implemented RNNs for traffic analysis, especially after the study in [10]. LSTM is specifically designed to memorize long-term dependencies [11]. Typically, an LSTM is formed by a chain of repeating memory blocks, each of them consisting of interactive neural network layers, as introduced in [50]. These layers are regulated by gating units to control the input/output information flow, enabling each block to update its memory. A more detailed description can be found in [10,50].

In traffic prediction, the temporal aspect (i.e., specified time horizon to predict traffic values) typically depends on the scope of the application (e.g., long- or short-term prediction) [3]. However, when developing a particular model, it is important to consider the spatial dependency across a road network [3,9]. To address this in a simple way, the study in [12] combined traffic data from multiple loops along a freeway and calculated an average traffic speed that was used as input for LSTM predictions. A more sophisticated way was developed by [51], transforming the traffic speeds of a road network into a 2D image where speed in each road segment was represented with different pixel values. These images were then used as input to a CNN alongside an LSTM model to capture the spatio-temporal dependencies for speed prediction. Alternatively, the study in [52] optimized an origin–destination (OD) matrix to spatially represent a traffic network capable of predicting how traffic propagates from one link to adjacent links with the aid of an ARIMA model. They used historic data wherever available, while traffic flows were simulated to fill data gaps along missing links. The aforementioned studies demonstrated that the spatial aspect in traffic prediction mainly relies on data availability, especially for a city-scale prediction application.

## 3. Methodology

### 3.1. Data Description

Tests were conducted at six CCTV locations in a part of the North East urban arterial road network in Newcastle upon Tyne and Gateshead, UK. CCTV raw datasets were retrieved from North East Combined Authority (NECA) Travel and Transport Data [53], UK. These datasets are freely available and, together with the CCTV locations, can be retrieved from the Urban Observatory (UO; [54]) API. The chosen CCTV cameras are located on roads with different classifications, such as A roads (e.g., A1058) and B roads (e.g., B1305), with various traffic conditions and volumes.

As CCTV cameras are set up by NECA operators to automatically switch views every few minutes, an image is captured from one of a number of different directions every time the camera turns. The number of views per CCTV differs. In addition, as every CCTV has a set of specific views, and vehicle counts of a single camera do not necessarily follow an identical pattern in all views (e.g., having city center surface parking in view provides high counts in off-peak hours). It should be noted that the CCTV cameras are categorized per location and not per view on NECA and UO web platforms. Due to this particular setup, the estimated vehicle counts have an irregular time interval and can often include gaps.

### 3.2. Workflow and Experiments

**Figure 1:**
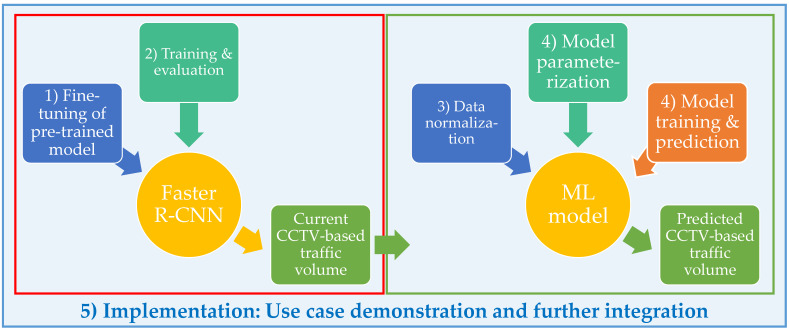
End-to-end CCTV-based traffic volume analysis and prediction.

The overarching end-to-end framework of the CCTV-based traffic volume analysis and prediction is schematically presented in Figure 1. CCTV-based vehicle counts detected with a fine-tuned Faster R-CNN constitute the current state of traffic volume. Those generated vehicle counts then feed the prediction analysis which can support decision making before, during and after a disruptive event via an online platform.

The methodological workflow of the framework is broken down into five stages, as follows: (1) Fine-tuning of pre-trained Faster R-CNN; (2) estimation of vehicle counts and post-training evaluation; (3) normalization of traffic volume data; (4) model parameterization, training and prediction; (5) implementation. The first two stages refer to vehicle count generation from CCTV images which characterize the traffic volume. Stage 3 prepares traffic datasets for model training and prediction. The final stage of implementation demonstrates a use case of the workflow for a six-day period. It also explores integrating the fine-tuned models for traffic volume detection and prediction on web-based platforms (e.g., Flood-PREPARED architecture [55]). Note the explanation of the integration is out of the scope of the presented work, and will be covered in a future study.

Regarding the first two stages, preliminary investigations were carried out in [30] to analyze the optimal deep learning neural network for vehicle detection in CCTV images with respect to precision and recall. The results showed that the fine-tuned Faster R-CNN model provided a better harmonic mean (F) (80%) than the fine-tuned SSD MobileNet, but with a relatively unsatisfactory recall (69%). Therefore, additional tests involving the development of a frozen inference graph model with improved performance in vehicle detection are carried out in this study.

In stage 4, three prediction approaches from parametric and nonparametric categories were tested, namely: SARIMAX, RF and LSTM. These were assessed under two different scenarios: Firstly using one-month (from midnight 1 July to midnight 2 August 2019) and four-month (from midnight 1 April to midnight 2 August 2019) periods of training/validation datasets and secondly including spatio-temporal time series from neighboring CCTV sensors via an OD matrix. To keep the consistency across all experiments for direct comparison, 10% of the experiment dataset served as a validation dataset. After identifying the optimal settings per prediction model, the models were re-trained using the entire dataset without omitting the 10%. In all experiments, final predictions were evaluated with respect to detected vehicles counts, on 2 August 2019 from 06.30 to 19.00, which constitute the “ground truth” test dataset. A final prediction assessment was also conducted over the period 3–9 August 2019 and included in stage 5 of the methodological workflow.

### 3.3. CCTV-Based Traffic Volume (Stages 1 and 2)

Additional tests using the same computing platform to those described in [30] assessed the performance of two model types with precision, recall and F, reported in Table 1. More information about the calculation of the aforementioned metrics can be found in [30]. The Faster R-CNN ResNet 101 and the Faster R-CNN Inception V2 are the two model types tested. Pre-trained frozen inference graphs of those types with COCO datasets were retrieved from [8]. The Faster R-CNN architecture consists of a convolution layer where a 600 × 1024 image is fed through, followed by a pooling, a fully connected and a softmax layer. A 0.7 and a 0.6 intersection over union (IoU) threshold was used in the initial and second stage of the model post-processing, respectively. The maximum number of region proposals was set at 300, the localization loss weight was equal to 2 and the classification loss weight was set to unity. Most parameters were configured as the default, based on the configurations of pre-trained models in [8]. A learning rate of 0.0002 and a 0.9 momentum optimizer were used during the model development with the different numbers of epochs per model indicated in Table 1. To fine-tune the pre-trained models, a total of 1269 NECA CCTV images from the year 2018 were used. Fifty NECA CCTV images which were not included in the training dataset were used as ground truth.

Any type of vehicle was manually labeled as a single class by two operators; the first one labeled 569 images and the second operator 700 images. After conducting six tests, as seen in Table 1, it was found that mixing the training images from two operators increased the number of false negatives with a low recall and harmonic mean. This may have been caused by unlabeled vehicles in the set of 700 images, resulting in false negatives. The Faster R-CNN Inception V2 model provided the highest number of identified vehicles and the best recall and harmonic mean among all tests.

**Figure 2:**
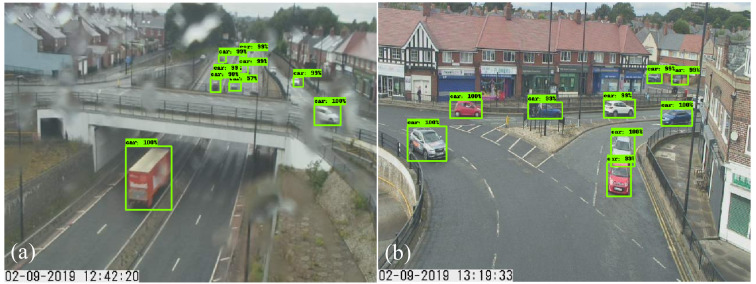
Detected vehicles on 2 August 2019 in two out of four views of the NC_A1058C1 CCTV. (**a**) View towards a main road; (**b**) View towards a residential street.

Figure 2 shows the vehicle detection results of the NC_A1058C1 CCTV camera from two different views. Figure 2a illustrates that nine vehicles were identified, with 19 missed because they were relatively small in size in the background of the image. Droplets on the CCTV sensor deteriorated the image quality. In Figure 2b, the fine-tuned model identified nine out of 10 vehicles from a second view of the NC_A1058C1 camera approximately 40 min later when the rain had stopped. It should be noted that the traffic volume estimated here incorporates vehicle counts from different lanes and directions, as CCTV cameras alter views (Figure 2). Moreover, the fine-tuned model required circa 0.4 s per image to detect and record the number of vehicles as well as export a single image, as in Figure 2. For that, an i5-6500 CPU@3.2GHz Ubuntu 16.04.5 LTS with a graphics processing unit (GPU) Quadro P4000 was used. The vehicle count time series generated with the Faster R-CNN fine-tuned model served as training, validation and “ground truth” test datasets in the experiments, presented here in stages 4 and 5.

### 3.4. Data Normalization (Stage 3)

Prior to any model training and prediction, data normalization (stage 3) is implemented, as follows:

(1) CCTV cameras with which prediction is planned to be modeled are designated as target cameras. Six target cameras are used in all experiments here, but the framework can be designed to select more CCTV cameras;

(2) To ensure regularity, time series are aggregated by calculating the average vehicle count over a time period. This aggregation period can overcome the challenge of varying camera views per location. The selection of an optimal aggregation period is described in stage 4;

(3) A filtering process flags those cameras with null counts for two consecutive days, excluding them from becoming target cameras, as they do not provide adequate observations for model training. The same filtering process excludes cameras with counts of an identical value for two consecutive days, to remove noise from the time series to be used as a training dataset;

(4) Filtered time series are reshaped to data formats suitable for SARIMAX, RF and LSTM algorithms, with respect to a specified aggregation period, the input exogenous attributes and a past sequence. The latter constitutes a time series in the past that can be used as input for predicting one step forward in the future.

**Figure 3:**
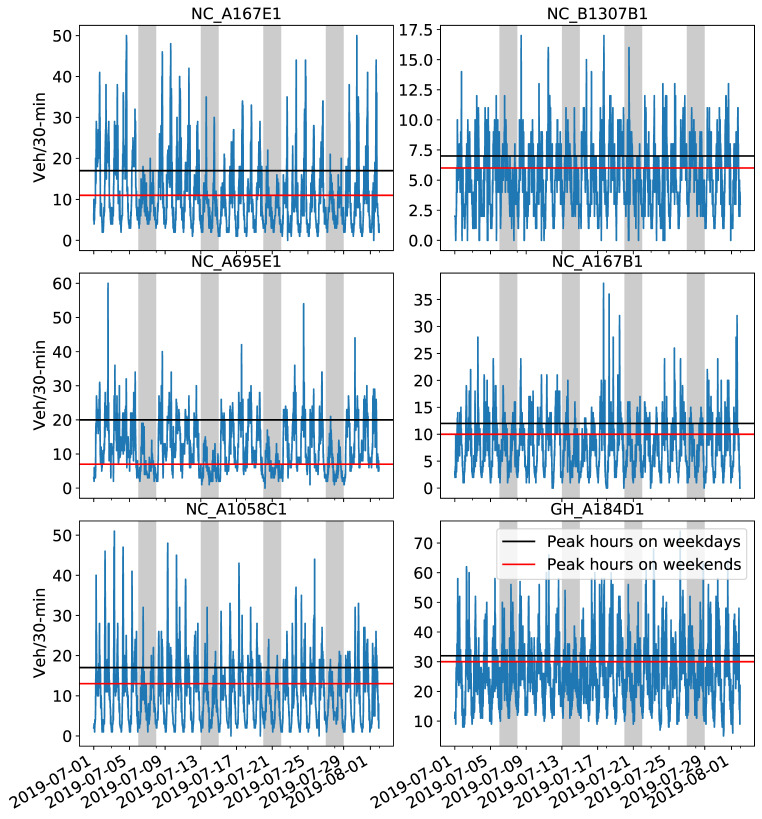
Aggregated vehicle counts showing the traffic volume during July 2019 (including 1 August 2019) at the six CCTV locations. Black and red lines represent the highest 25% of traffic volume on weekdays and weekends, respectively. Gray zones indicate weekends. Note the different scales of the y-axes.

One month of traffic volume was used for training (Figure 3). The 25% highest flow on weekdays and weekends is equivalent to the 3rd quartile of the July 2019 dataset. Intuitively, this indicates variations in traffic behavior during peak hours due to people driving from/to work. Because the chosen CCTV sensors represent traffic volume for roads of different classifications, the number of vehicles varies, as seen on the y-axes in Figure 3. For instance, the NC_B1307B1 CCTV showed the lowest variation between weekends and weekdays compared to the NC_A695E1 CCTV (Figure 3). Similarly, different low and high peaks were observed at the six CCTV sensors in a single day. To accommodate such daily and weekly temporal patterns in the prediction models, four exogenous factors were added in the input time series as follows: (1) Weekend or weekday; (2) day of the week; (3) period of the day before; and (4) after midnight.

### 3.5. Evaluation Metrics for Prediction

Three error metrics were adopted to assess the prediction models’ performance, namely, (a) mean absolute error (MAE); (b) the mean absolute percentage error (MAPE); and (c) the root mean square error (RMSE). These were calculated as follows:

(1) $$\mathrm{MAE}=\frac{1}{N}\sum_{i=1}^{N}\left|x_{i}-y_{i}\right|$$

(2) $$\mathrm{MAPE}=\frac{1}{N}\sum_{i=1}^{N}\left|x_{i}-y_{i}\right|$$

(3) $$\mathrm{RMSE}=\sqrt{\frac{1}{N}\sum_{i=1}^{N}{\left(x_{i}-\;y_{i}\right)}^{2}}$$

where *N* is the length of evaluation data and *x_i_* is the measured and *y_i_* the predicted value of the *i^th^* observation. It should be noted that no issue with division by zero in (1) was encountered, as vehicle counts were always greater than zero during traffic hours (06.30–19.00) for the test dataset.

### 3.6. Prediction Model Parameterization (Stage 4)

To identify optimal parameters per model before conducting the actual experiments, a tuning process was carried out using 90% of the one-month (July 2019) datasets as training data and 10% as validation data.

The tuning process for RF model development involved the setting of four parameters as follows: Number of trees; maximum depth of a tree; minimum number of samples to split an internal node of a tree; and minimum number of samples at the leaf node (i.e., the tree base), as defined in the Python scikit-learn library [56]. The tuning process was applied to the one-month time series of the NC_A167E1 CCTV (Figure 3). The process also examined the RF prediction performance using different hours and minutes for past sequence and aggregation periods, respectively, as seen in Table 2. To estimate the best combination of parameters, a grid search algorithm was adopted using a 3-fold cross validation strategy in GridSearchCV [57] calculating the MAE, as reported in Table 2. The 3-fold strategy performs cross-validation after fitting a number of RF models equivalent to the number of candidates (i.e., various combinations of parameters) multiplied by three. For instance, 9600 candidates were cross validated three times for tuning with 30 min aggregated training data, which required 10 h on an i5-6500 CPU@3.2GHz Ubuntu 16.04.5 LTS. MAPE and RMSE metrics were calculated against the 10% validation dataset of the one-month time series.

As evidenced in Table 2, all evaluation metrics became minimum when data were aggregated over 60 min, regardless of the given past sequence. However, such a period smoothed the traffic pattern and disregarded subtle traffic behavior that occurred within an hour. In contrast, due to higher temporal resolution, a 15 min aggregation period could incorporate abrupt changes in traffic behavior, but with high MAE and RMSE metrics close to unity in most cases. To overcome this, a 30 min aggregation period was considered as the trade-off between performance and the sufficient capture of unexpected short-term traffic behavior. This time period was also considered suitable as it could accommodate the challenges of varying views in the CCTV images. For the 30 min aggregation period, all three metrics were lower when 12 and 24 h past sequences were used. Intuitively, when a longer past sequence is fed into a model, the prediction can be more stable. Hence, a past sequence of 24 h and a 30 min aggregation period were selected for all experiments. Based on those variables, the tuning process was repeated for the remaining five CCTV locations. Afterwards, the input time series, together with the exogenous factors, were reshaped into a one-dimensional array and fed into the RF regressor for training using the tuned parameters reported in Table 3.

Regarding the tuning process for SARIMAX, there are seven parameters to specify. The model is typically denoted as SARIMAX (p, d, q) × (P, D, Q) [S] where: S refers to the number of periods per season; p is the autoregressive term expressing the number of lagged observations; d is the integrated term indicating the degree of differencing of raw observations; q is the moving average term; and uppercase P, D and Q are the equivalent autoregressive, integrated and moving average terms of the model’s seasonal part. More details on the SARIMAX model can be found in previous studies [35,39,40,42]. An inspection of the time series for the year 2019 ensured that there was no apparent downward or upward trend in the datasets with respect to the four annual seasons. However, as evidenced in Figure 3, a daily seasonal pattern was observed, implying that S is 24 h, equivalent to 48 for a 30 min aggregation period per day. Similar to [35], since there was no trend in the datasets, adopting S = 48 ensured that the time series became stationary with a lag (i.e., order of difference) equal to one day. This is in line with the selected past sequence of 24 h, as explained previously.

As also suggested in [42], the autoregressive and partial autoregressive correlation function plots were utilized to define a range of candidate values (between 0 and 5 for the six seasonal and non-seasonal parameters). A grid search algorithm was adopted as found in the statistical “pyramid” Python library [58]. This uses Akaike’s information criterion (AIC) to identify the optimal set of SARIMAX parameters with the best fit to the provided datasets (for AIC calculation, see also [35]). The grid search was applied to the datasets of the six CCTVs and the best combinations of parameters are listed in Table 4.

Regarding the LSTM model development, various tests for tuning hyperparameters were carried out using the one-month time series for the NC_A167E1 CCTV camera. Initially, based on trial and error, two LSTM layers using the ReLU activation function were found to be suitable, with a single dense layer and a single dropout layer with a linear activation function following afterwards. The Adam optimizer was adopted with a learning rate set at 0.001 and the mean squared error used as the loss function during training. The one-month time series data were reshaped into a three-dimensional array as required for training and validation with a 128 batch size. For instance, a validation dataset with a length of 140 observations (i.e., 10% of the one-month dataset) was reshaped into (90,49,5) where 90 is the number of data samples, 49 the length of the past data sequence including the last 30 min time step (i.e., a window of 24 h) and 5 the number of input attributes including the time series of the NC_A167E1 camera alongside the four exogenous factors.

**Figure 4:**
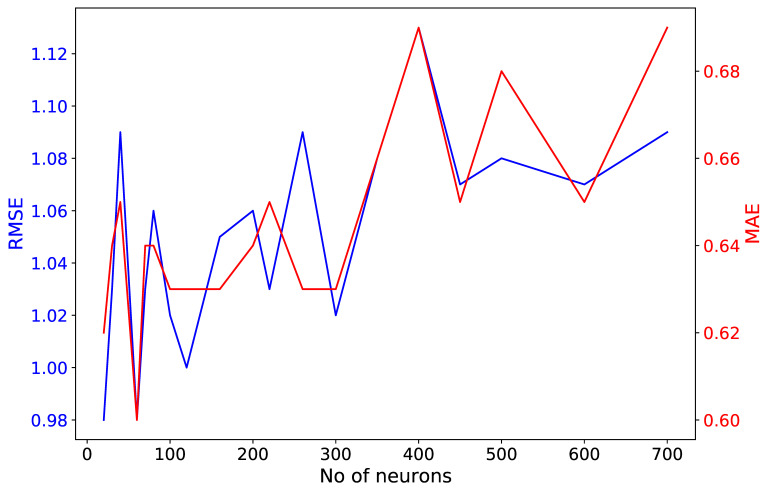
MAE and RMSE metrics estimated using the validation dataset within a grid search test under different numbers of long short-term memory (LSTM) neurons.

An additional test investigated the optimal number of neurons of the two LSTM layers, applying a grid search for a range of [10–700] neurons run for 100 epochs. MAE, MAPE and RMSE were calculated with respect to the measured traffic volume of the validation dataset. As seen in Figure 4, there was no significant increase in the metrics’ magnitude when hundreds of neurons were set for the two LSTM layers. However, a slight upward trend was observed for MAE and RMSE values. MAPE was not plotted in Figure 4 because its magnitude did not differ more than ±0.02 from a value equal to 0.21. Based on this test, 60 neurons were chosen as units in the LSTM layers, as they provided minimal values for all three metrics (MAPE = 0.20, MAE = 0.60 and RMSE = 0.98).

**Figure 5:**
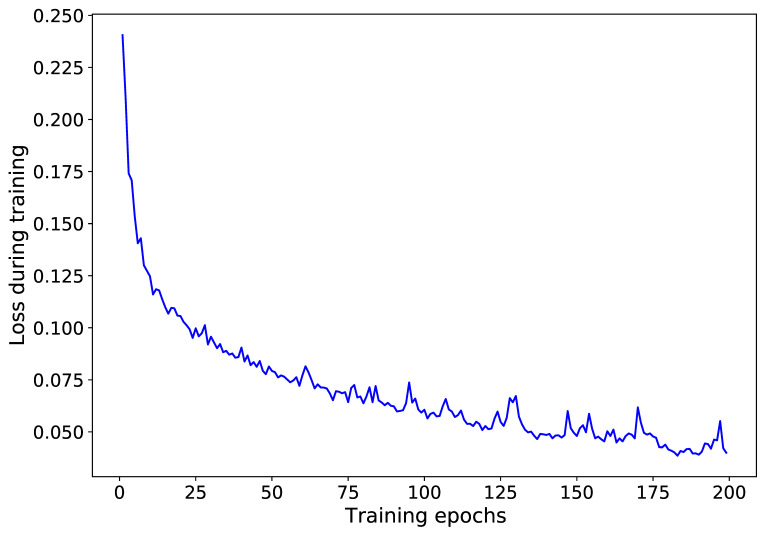
Loss curve during training with the NC_A167E1 one-month dataset.

It should be noted that for the LSTM model development and prediction, a Python script was retrieved from [27], and was amended accordingly. The script utilizes Tensorflow and Keras routines for LSTM modeling. After defining the LSTM structure, six models were trained for 200 epochs, separately, per CCTV location with the input being all the observations for one- and four-month datasets per experiment. With a trial and error procedure, it was found that 200 epochs were sufficient, resulting in a low loss value. Overfitting was examined by monitoring the loss of the validation dataset during the aforementioned tests for the LSTM structure development. An example of a loss curve for the NC_A167E1 CCTV camera is shown in Figure 5. The algorithm picks the training epoch with the lowest loss value to build the LSTM model.

Regarding time consumption for model training, approximately 4 min and 15 min per CCTV sensor were required for a 200-epoch training of an LSTM model with one month and four months of data, respectively. The tests were conducted with a GPU Quadro P4000 in Ubuntu 16.04.5 LTS. Without the use of a GPU, approximately 1.2 and 4.7 min were required to train an RF model with one month and four months of data, respectively.

### 3.7. Origin–Destination (OD) Matrix

**Figure 6:**
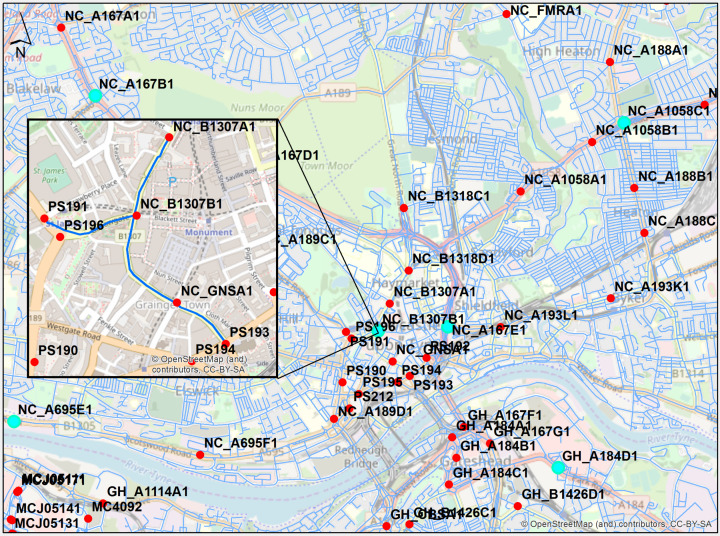
Part of the structured arterial road network in Newcastle upon Tyne and Gateshead shown with blue lines with target CCTVs illustrated in cyan. Inset map depicts the shortest routes in blue, whose lengths are stored in the origin–destination (OD) matrix. These routes were used to select the four closest cameras to the NC_B1307B1 CCTV.

To incorporate spatial dependencies from neighboring CCTV sensors into traffic predictions, an OD matrix was firstly structured across the 219 CCTV locations in the North East region. This was achieved with the aid of the network analyst tool in ArcGIS from ESRI [59]. An arterial road network model was built using A and B roads supplied by Ordnance Survey [60]. A part of the road network is mapped, shown with blue lines, in Figure 6.

The OD matrix was constructed based on the calculated shortest routes along the road network between all CCTV locations. This was then imported into the data preparation step to select traffic data from the four closest CCTV sensors that are as evenly distributed as possible across the north, east, south and west directions from the target camera. Prior to this selection step, the traffic data were filtered out from the noise, as described in stage 3: Data normalization. The filtered time series of traffic data from the selected nearby CCTV sensors was considered additionally to the four exogenous attributes. The selection process is automated and follows three steps as below:

(1) Select a camera closest to pre-defined distance from target cameras (0.20 m was used in the experiments here);

(2) Calculate ideal bearings to cameras based on the desired number of cameras to include in the final training set (e.g., if 4 cameras are to be selected, then the angle between them should be 360/4 = 90 degrees) The bearing is calculated based on the aforementioned angle (e.g., when the first bearing is at 45 degrees from the target camera, then the other three bearings would be 135, −135 and −45 degrees);

(3) For every bearing left, rank all candidate cameras used for training except the first selected (in step 1) based on how close they are to the desired distance and how close they are to the desired bearing and apply weights to choose the best-scoring camera. Distances and bearings are normalized between 0 to 1 and expressed as below:

(4) $$Normalized_{dist\_bearing}=d^{Wd}\cdot b^{Wb}$$

where *d* and *b* are the calculated distances and bearings between candidate CCTVs with *W_d_* and *W_b_* as their corresponding weights. Here, weights of 4 and 1 are used for distance and bearing, respectively. These values were chosen after a trial and error process as they seemed most suitable to identify the closest cameras to the target one.

The inset map in Figure 6 shows an example for the NC_B1307B1 target camera with PS191, NC_B1307A1, NC_GNSA1 and PS193 as the assigned four nearby CCTV cameras for the one-month time series. It should be noted that the PS196 camera included null traffic data for the specified training periods, hence, it was excluded from the selection step, even though it was the closest to the target camera, among others. Additionally, there were no CCTV cameras located in the east direction close enough to be selected, therefore the PS193 camera was considered as the fourth choice.

## 4. Results

### 4.1. Resulting Predictions with Zero Nearby CCTVs

**Figure 7:**
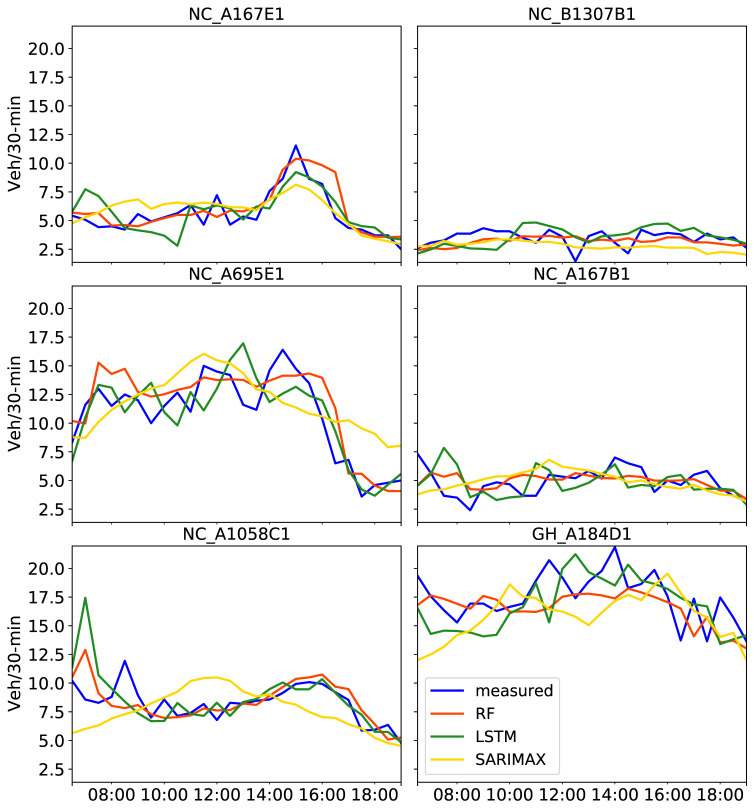
Predictions on 2 August 2019 obtained with three tuned models, RF, LSTM and SARIMAX, using the past one month of training data including four exogenous attributes. Measured traffic volume constitutes the “ground truth”.

Predicted traffic volumes between 06:30 and 19:00 on 2 August 2019 are shown in Figure 7 and the corresponding evaluation metrics are reported in Table 5.

These results refer to the prediction models built for each target camera with four exogenous attributes not including any neighboring CCTV cameras. As clearly evidenced in Figure 7, SARIMAX predictions provided the worse results among the three models, as traffic did not follow the actual patterns. For instance, for NC_A1085C1, SARIMAX predicted a high peak at around midday, whereas the high peak was observed at 16:00. This is also reflected in Table 5, with SARIMAX MAE and RMSE values being greater than unity at all CCTV locations but the NC_B1307B1 camera.

Similar results were observed with MAE and RMSE values calculated from LSTM predictions, possibly because the flow in this CCTV location did not include high variations and the predictions from all three models could capture the relatively constant traffic behavior. When comparing LSTM and RF in Figure 7, distinctive discrepancies are observed. For instance, LSTM underestimated the traffic volume in the morning for the GH_A184D1 camera, predicting a low peak when the actual traffic showed a high peak. RF also failed to capture the sudden peaks at this location. As reported in Table 5, estimated MAE and RMSE values were the highest for this camera compared to the others for both LSTM and RF. However, RF provided the best overall performance based on the average values for the three evaluation metrics in Table 5.

**Figure 8:**
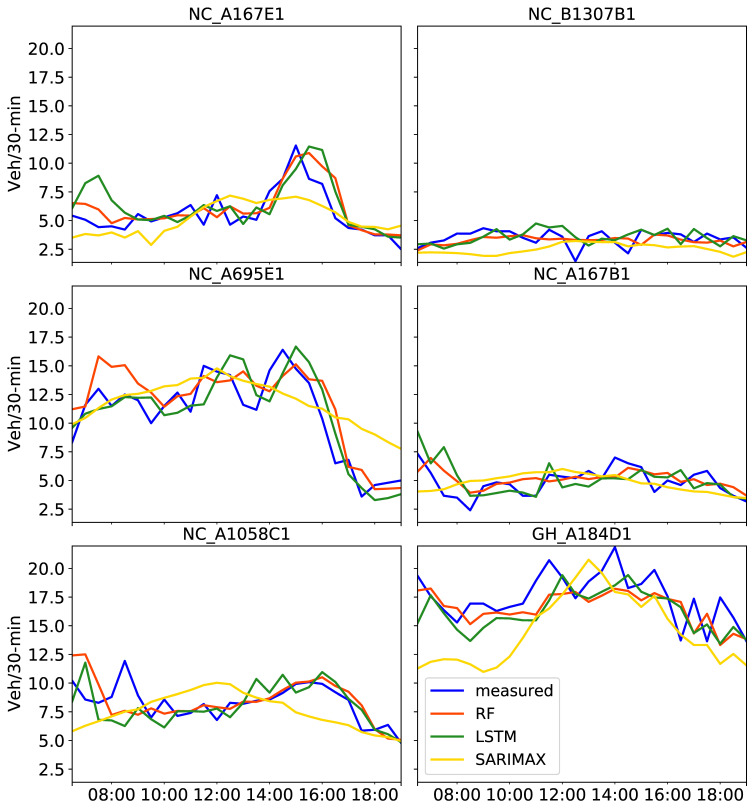
Predictions on 2 August 2019 obtained with three tuned models, RF, LSTM and SARIMAX, using the past four months of training data including four exogenous attributes. Measured traffic volume constitutes the “ground truth”.

Regarding the overall performance of the predictions (i.e., average values in Table 5) when compared to the amount of training data, metrics were worse for SARIMAX, better for LSTM and consistent for RF results. As also seen in Figure 8, no apparent improvement of the predicted traffic curves were observed with SARIMAX. The difference between Figure 7 and Figure 8 is that the latter shows predictions after training with a four-month dataset. The wrong peaks LSTM predicted in the morning at NC_A1058C1 and GH_A184D1 seen in Figure 7 were considerably improved in Figure 8. This is reflected in Table 5, with lower RMSE values for the four-month compared to the one-month training datasets at those CCTV locations. When comparing LSTM with RF results in Table 5, RF MAPE values were improved for the four-month training at all locations with the exception of two CCTV cameras (NC_A167E1 and NC_A695E1), but all were lower than 0.20. Overall, RF models using either one month or four months of training provided more consistent predictions. However, the four-month training period clearly improved LSTM predictions in most cases. As noted in [45], the longer the training is, the more context the models can incorporate, in turn resulting in reduced prediction errors.

### 4.2. Resulting Predictions with Four Nearby CCTVs

**Figure 9:**
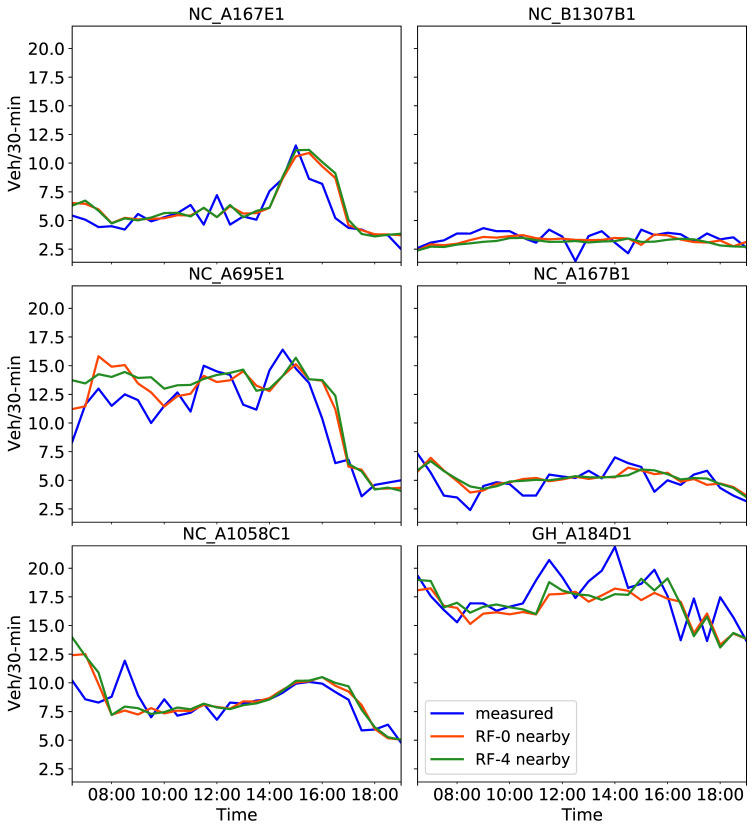
Predictions on 2 August 2019 obtained with the RF tuned model using the past four months of training data including four (line in red) and nine exogenous attributes, four of which refer to four nearby cameras (line in green). Measured traffic volume constitutes the “ground truth”.

**Figure 10:**
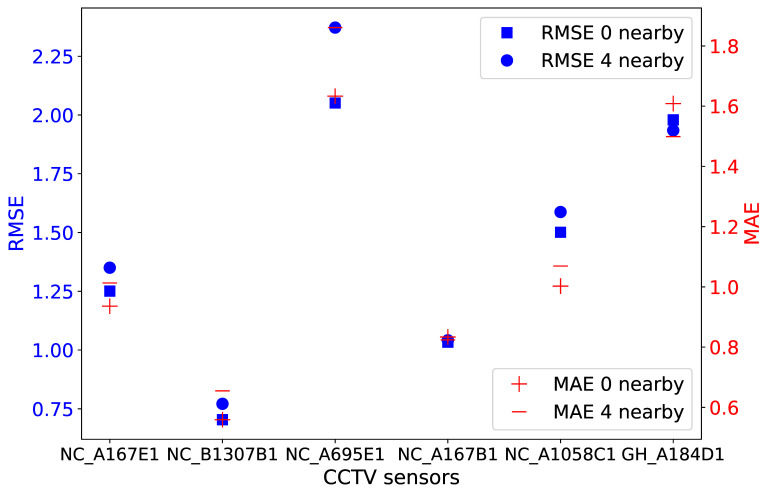
MAE and RMSE values calculated per CCTV sensor for RF prediction results shown in Figure 9. Values in blue and red correspond to RMSE and MAE, respectively.

Investigations presented hereafter used the RF prediction model, which showed better consistency than all other models in the previous experiments. Traffic predictions on 2 August 2019 from the experiment including four neighboring CCTV sensors based on the OD matrix are shown in Figure 9. Prediction models were built using four-month training datasets and nine exogenous factors. The additional five factors included the time series of the four closest CCTVs to the target camera (e.g., in Figure 6). Calculated MAE and RMSE values are illustrated in Figure 10.

By inspecting the predicted traffic volume in Figure 9, a few variations between the two results were observed only for the NC_A695E1 and GH_A184D1 sensors. For instance, the inclusion of traffic from neighboring locations during modeling improved the prediction of high peaks at the GH_A184D1 camera at midday. Other than that, RF provided a consistent prediction regardless of the use of traffic from neighboring locations. This finding is also depicted in Figure 10, where RMSE and MAE variations lie within a maximum of 0.32 and 0.23, respectively, both at the NC_A695E1 location. MAPE values, estimated after taking into consideration four nearby cameras, are not reported here as they did not differ from those values in Table 5 by more than ±0.02, with an average 0.17 for all sensors.

### 4.3. Implementation (Stage 5)

#### 4.3.1. Use Case Demonstration

**Figure 11:**
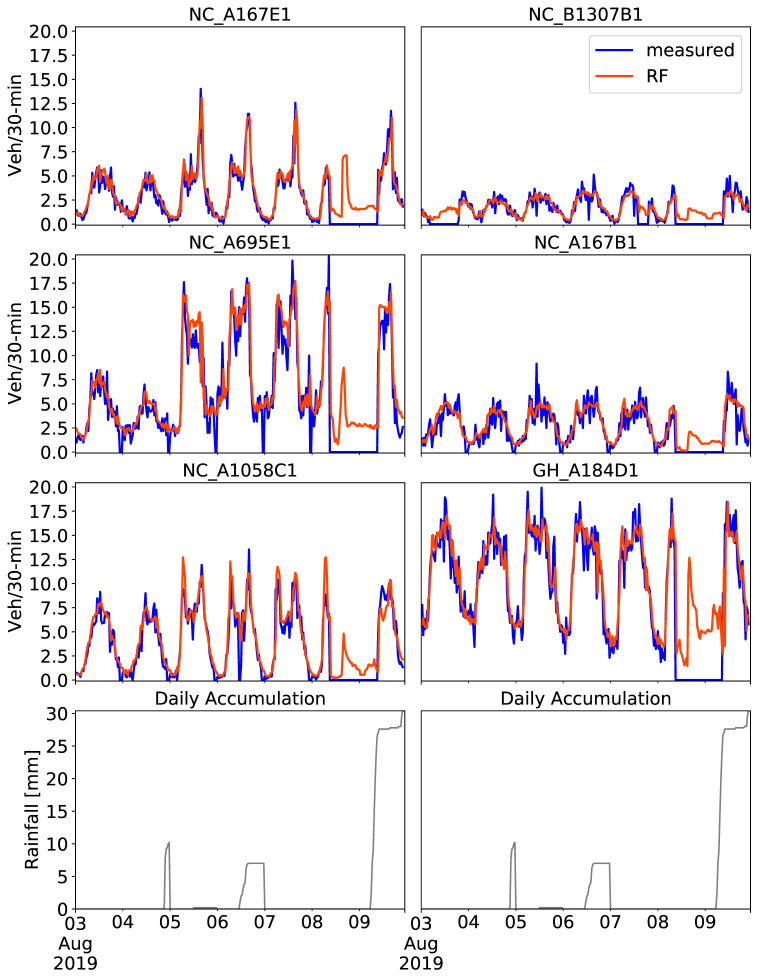
Predictions on 3–9 August 2019 obtained with the RF tuned model using the past four months of training data including nine exogenous attributes, four of which refer to four nearby cameras. Measured traffic volume constitutes the “ground truth”. Daily accumulated rainfall is illustrated in gray.

The end-to-end CCTV-based traffic prediction framework was implemented in a six-day period at the six CCTV locations. Predictions were estimated based on the RF models fine-tuned on four-month training datasets alongside nine exogenous attributes using four nearby CCTV locations. The results for 3–9 August 2019 are depicted in Figure 11, together with the daily accumulated rainfall, as recorded by a weather station located in Newcastle upon Tyne city center [61].

That week, a lot of missing data were observed. Data gaps are caused by a possible sensor malfunction (e.g., abrupt shutdown of the sensor), but are often due to poor weather conditions. Vehicle counts were not recorded mostly during rain on the morning of 9th of August, hence, the zero values shown in blue. Data gaps were also observed at NC_B1307B1 on 3rd of August, but not because of the poor weather in this case, as there was zero rainfall observed. However, RF predictions were able to provide a continuous time series of traffic conditions for the whole week with a sufficient overall performance. In a few cases, RF predictions overestimated abrupt zero peaks, as seen at NC_A695E1, for example. On the other hand, abrupt high traffic peaks were correctly captured at NC_A167E1, NC_A695E1 and NC_A1058C1, 50% of the CCTV locations.

Due to missing data, there are not sufficient ground truth references to calculate evaluation metrics in this case. However, the results in Figure 11 highlight the potential use of the CCTV-based RF prediction framework even with the traffic data gaps. As explained in stage 4—Prediction model parameterization, 24 h of traffic data was used as the past sequence for the vehicle count prediction for the following 30 min. Even though there was a long data gap between 8th and 9th of August, the RF could still predict the traffic volume during the evening on 9th of August with sufficient level of accuracy. A few discrepancies between the measured and the predicted values can be seen at NC_A695E1 and NC_A1058C1 around sudden traffic peaks. This investigation has provided confidence that the presented CCTV-based traffic RF prediction constitutes an effective end-to-end framework with practical usage and simple implementation.

#### 4.3.2. Integration with Online Platforms

Integration of the framework is explored on two online platforms: The UO and the Flood-PREPARED project [62]. Regarding the current traffic volume, the frozen inference graph of the Faster R-CNN model developed in the presented study is currently running on the UO platform for vehicle detection. Time series of vehicle counts are stored by the UO and updated daily and are freely available through the UO API. Regarding traffic predictions, the RF prediction results at NC_A167E1 are currently visualized on the Flood-PREPARED dashboard [55] as a single past event (https://www.nismod.ac.uk/fp/traffic_disruption). The Flood-PREPARED framework is currently under development though it aims to provide a warning mechanism for fast-evolving, short, extreme rainfall events and visualize the impacts of such events, including on traffic in Newcastle upon Tyne city center. The Flood-PREPARED dashboard is intended to: (a) Retrieve real-time series of CCTV-based vehicle counts from UO; (b) store the fine-tuned RF prediction model, which would be updated on a two-week basis; (c) predict and visualize traffic on specified time horizons in real time (e.g., for the next three hours, or for daily and weekly peak hours).

## 5. Discussion

In terms of assessing three different prediction approaches, the presented experiments have demonstrated that the SARIMAX model failed to accurately capture the traffic conditions in all six CCTV locations, despite the ease of its implementation. However, LSTM and RF provided predictions closer to the “ground truth” data but required demanding preparations to implement their algorithms. With regard to computational efficiency, RF does not require GPU and runs faster compared to LSTM. This is advantageous for the deployment in real-time visualization platforms such as the Flood-PREPARED architecture/web-based dashboard. Currently, the RF algorithm can build multiple prediction models for different locations at once, when the model parameters per CCTV sensor are identified in a previous step. Moreover, future work to include weather conditions, social events and holidays as additional exogenous attributes will be investigated. This would enable traffic volume predictions to ultimately be associated with more realistic contextual information, providing a better understanding of the impact on the city’s road infrastructure before, during and after a disruptive event.

Regarding the three predictors’ performance, RF delivered the most consistent results across various experiments. The inclusion of a four-month time series for training significantly improved LSTM predictions. In addition, RF predictions became more accurate, especially in capturing traffic peaks. The further inclusion of detected vehicle counts from four neighboring CCTV locations showed a general consistency in RF outputs and the model worked sufficiently well when missing data were observed due to CCTV camera shutdowns. A combination of other approaches, such as KNN, with the OD matrix in the RF model, could potentially improve the current selection process of nearby CCTVs and include only correlated spatio-temporal information in the traffic prediction. Overall, it was shown that the RF machine learning method constitutes a simple and fast method for a real-time application, requiring less computational demand, while also not requiring a GPU as in other deep learning methods.

Regarding the overall prediction accuracy, a validation process was applied at two different stages of the framework. Firstly, the vehicle detection outcome, modeled with the fine-tuned Faster R-CNN, was evaluated with 50 NECA CCTV images, in which manually identified vehicles were used as ground truth. This is a common procedure for object detection evaluation, while the ideal scenario would be to have a number of vehicles as ground truth measured by other means, such as inductive loops. This setup was not feasible at the time the experiments took place. However, after experimentation with different numbers of images and epochs for training, the fine-tuned Faster R-CNN Inception V2 model provided a harmonic mean greater than 90% with the highest number of detected vehicles (Table 1). This was indicative of a successful vehicle detection performance, sufficient to be used for the next phase of prediction. A second validation step was then applied to evaluate predicted values of the three approaches based on results produced by the chosen fine-tuned Faster R-CNN Inception V2 model. That way, even in the case of a poor vehicle detection result, the following prediction accuracy would still be high, under the condition of a well-performed prediction algorithm like the RF or LSTM models. In the case of the SARIMAX model, a poor prediction would not be attributed to the low vehicle detection accuracy but to the underperformance of the prediction model itself. In other words, a poor vehicle detection accuracy does not adversely affect the overall prediction outcome in the developed framework.

Regarding future directions, the end-to-end prediction framework can be potentially implemented at any CCTV location on the region’s IoT infrastructure, providing a useful tool for the traffic management bureau. Through providing real-time data to decision makers prior and during a disruptive event (e.g., flood) it could enable more effective responses to such events. To that end, the integration with a web-based visualization platform such as the Flood-PREPARED dashboard will allow users to easily access up-to-date data on current traffic volume as well as predictions ahead of time, along with other contextual information, providing detailed data for city traffic managers, emergency planners and others responding to an event to better inform response decisions. It should be mentioned that for real-time web-based applications, it is important to find the balance between prediction accuracy, computational capacity and cost. Hence, a simple machine learning method could be implemented more easily than a more complex deep learning method provided that accuracy levels are sufficient and the web-based integration follows a low-cost solution to ensure the longevity of the real-time application.

## 6. Conclusions

The research presented here has demonstrated an end-to-end practical machine learning framework for CCTV-based urban traffic analysis and prediction. The framework has exploited the open-source pre-trained Faster R-CNN models to generate vehicle count series across the arterial road network in the North East region, UK, which are now freely available to the public through the UO API. The framework has investigated the performance of three approaches (SARIMAX, RF and LSTM) to predict traffic volume based on a vehicle count time series. The results have shown that RF and LSTM outperform SARIMAX, with RF providing consistency and time efficiency across all experiments. The entire framework has been demonstrated in the use case of a six-day period with missing data, showing that the RF predictions were sufficiently accurate across all six CCTV locations. Overall, it can be concluded that CCTV image series can be used for both vehicle detection and the prediction of traffic conditions with a great level of confidence to support the development of cost-effective real-time traffic management infrastructure.

## Figures and Tables

**Table 1:** Faster R-CNN post-training evaluation of 50 ground truth images.

Model	No of Images /Epochs (K)	Identified Vehicles	True Positives	False Positives	False Negatives	Precision (%)	Recall (%)	F (%)
Faster R-CNN ResNet 101	1269/26	326	318	8	74	97.5	81.1	88.6
Faster R-CNN ResNet 101	1269/164	330	320	10	77	97.0	80.6	88.0
Faster R-CNN Inception V2	1269/40	319	307	12	85	96.2	78.3	86.4
Faster R-CNN Inception V2	1269/91	318	308	10	84	96.9	78.6	86.8
Faster R-CNN Inception V2	569/200	374	355	19	42	94.9	89.4	92.1

**Table 2:** Optimal random forest (RF) parameters after grid search with evaluation metrics.

Agg. Period (min)	No. of Trees	Max. Depth of a Tree	Min. No. of Samples to Split	Min. No. of Samples at Leaf	MAE/MAPE/RMSE
Past sequence: 2 h					
15 min	105	55	5	5	0.99/0.27/1.08
30 min	115	100	8	5	0.86/0.20/0.94
60 min	93	75	6	2	0.84/0.12/0.64
Past sequence: 6 h					
15 min	134	80	5	2	0.98/0.18/0.77
30 min	115	90	7	5	0.86/0.17/0.85
60 min	121	55	8	7	0.84/0.15/0.79
Past sequence: 12 h					
15 min	127	95	5	4	0.97/0.21/0.90
30 min	105	30	10	3	0.86/0.15/0.76
60 min	112	45	5	3	0.87/0.11/0.61
Past sequence: 24 h					
15 min	96	40	10	4	0.95/0.21/0.94
30 min	96	25	10	4	0.82/0.15/0.75
60 min	90	45	7	3	0.80/0.11/0.68

**Table 3:** RF tuned parameters per CCTV after grid search.

CCTV	No. of Trees	Max. Depth of a Tree	Min. No. of Samples to Split	Min. No. of Samples at Leaf
NC_A167E1	96	25	10	4
NC_B1307B1	143	30	5	4
NC_A695E1	140	50	10	2
NC_A167B1	118	21	10	5
NC_A1058C1	96	48	7	3
GH_A184D1	131	48	10	2

**Table 4:** SARIMAX tuned parameters per CCTV after grid search.

CCTV Location	SARIMAX Parameters
NC_A167E1	(2.0,1) × (2,1,2) [48]
NC_B1307B1	(1.0,1) × (0,1,1) [48]
NC_A695E1	(1.0,1) × (1,1,1) [48]
NC_A167B1	(2.0,2) × (1,1,2) [48]
NC_A1058C1	(2.0,2) × (2,1,1) [48]
GH_A184D1	(1.0,1) × (0,1,1) [48]

**Table 5:** Evaluation metrics per CCTV per prediction model 06:00–19:00 2 August 2019.

CCTV Location	SARIMAX	LSTM	RF
	MAE/MAPE/RMSE	MAE/MAPE/RMSE	MAE/MAPE/RMSE
With past one month of training data			
NC_A167E1	1.04/0.19/1.27	1.07/0.21/1.35	0.86/0.16/1.19
NC_B1307B1	0.84/0.25/0.96	0.83/0.28/1.01	0.63/0.22/0.78
NC_A695E1	2.40/0.31/2.83	1.80/0.17/2.21	1.59/0.17/1.92
NC_A167B1	1.08/0.23/1.36	1.18/0.26/1.56	0.87/0.20/1.15
NC_A1058C1	1.85/0.22/2.21	1.22/0.14/2.11	0.91/0.11/1.38
GH_A184D1	2.50/0.14/2.99	1.92/0.11/2.39	1.67/0.09/2.11
Average value	1.62/0.22/1.94	1.34/0.19/1.77	1.09/0.16/1.42
With past four months of training data			
NC_A167E1	1.34/0.25/1.60	1.38/0.25/1.76	0.94/0.17/1.25
NC_B1307B1	1.12/0.33/1.27	0.66/0.23/0.84	0.56/0.19/0.70
NC_A695E1	2.09/0.29/2.55	1.53/0.16/1.81	1.63/0.18/2.05
NC_A167B1	1.03/0.23/1.31	1.00/0.22/1.32	0.83/0.19/1.03
NC_A1058C1	1.73/0.20/2.11	1.21/0.14/1.70	1.00/0.12/1.50
GH_A184D1	3.24/0.18/3.79	1.71/0.10/2.16	1.61/0.09/1.98
Average value	1.76/0.25/2.10	1.25/0.18/1.60	1.10/0.16/1.42

## Data Availability

Time series of vehicle counts are freely available through Newcastle Urban Observatory at http://uoweb3.ncl.ac.uk/.
